# The Use and Efficacy of Oral Phenylephrine Versus Placebo Treating Nasal Congestion Over the Years on Adults: A Systematic Review

**DOI:** 10.7759/cureus.49074

**Published:** 2023-11-19

**Authors:** Jaqueline Livier Castillo, Jose R Flores Valdés, Maria Maney Orellana, Sruthi Satish, Chimaobi E Ijioma, Janet Benjamin, Elisa Ramirez Alvarez, Marily Martinez Ramirez, Victor Sebastian Arruarana, Ernesto Calderon Martinez

**Affiliations:** 1 Internal Medicine, Universidad Autonoma de Guadalajara, Guadalajara, MEX; 2 Internal Medicine, Universidad de Carabobo, Valencia, VEN; 3 Internal Medicine, Kasturba Medical College, Manipal, Manipal, IND; 4 Medicine and Surgery, Abia State University Faculty of Medicine, Uturu, Umuahia, NGA; 5 Internal Medicine, Ross University School of Medicine, Miramar, USA; 6 Internal Medicine, Universidad Nacional Autonoma de Mexico, Mexico City, MEX; 7 Internal Medicine, Brookdale University Hospital and Medical Center, New York, USA; 8 Biomedical Informatics, Universidad Nacional Autónoma de México, Mexico City, MEX

**Keywords:** nasal airflow, sinusitis, seasonal, allergic, rhinitis, allergic rhinitis, oral, nasal decongestant, nasal congestion, phenylephrine

## Abstract

Nasal congestion is a common issue stemming from various factors such as allergies and anatomical variations. Allergic rhinitis frequently leads to nasal congestion. The pathophysiology involves inflammation, swelling, and mucus production in the nasal mucosa. Multiple treatments are available, including oral phenylephrine, an over-the-counter or prescription option. However, the effectiveness and safety of phenylephrine have been subjects of debate. This systematic review aims to provide an updated perspective on the efficacy of oral phenylephrine versus placebo in addressing nasal congestion in adults. We conducted following the Preferred Reporting Items for Systematic Reviews and Meta-Analyses (PRISMA) 2020 guidelines, a systematic review involving searches on PubMed, Cochrane, and Scopus databases. Inclusion/exclusion criteria were defined to identify high-quality studies. The focus was on randomized controlled trials (RCTs) and case-control studies published in English between 1998 and 2023, involving adult populations. The interventions compared oral phenylephrine with placebo or standard care, with outcomes centering on changes in nasal congestion symptoms and nasal airway resistance. We identified four articles that met the criteria. These studies exhibited varied designs and populations. The findings consistently indicated that phenylephrine was not more effective than a placebo in relieving nasal congestion. This systematic review demonstrates that oral phenylephrine did not offer substantial relief from nasal congestion compared to a placebo in adults. The studies featured diverse designs, yet the prevailing conclusion was that phenylephrine's efficacy was limited. Safety assessments showed no life-threatening adverse events, with common side effects including headaches and mild discomfort. In summary, this systematic review indicates that oral phenylephrine is not significantly more effective than a placebo in alleviating nasal congestion in adults. Clinicians should explore alternative treatment options, considering the review's limitations. Additional research may be needed to clarify the role of oral phenylephrine in managing nasal congestion.

## Introduction and background

Nasal congestion is a common symptom in clinical practice; it is the blockage of the nasal cavity, hindering proper airflow. It can be caused by reversible or inflammatory causes, such as allergic and non-allergic rhinitis, leading to acute or chronic inflammation of the nasal mucosa. Irreversible and constant nasal congestion could be caused by occlusion and anatomy variations such as nasal polyps, foreign body, turbinate hypertrophy, or septal deformity [1]. Allergic rhinitis (AR) is a common cause of nasal congestion, affecting 10% to 20% of the global population and 11.9% to 30.2% of adolescents and adults in the United States [2-4].

The pathophysiology of nasal congestion involves several mechanisms contributing to inflammation, swelling, and mucus production in the nasal mucosa. These mechanisms include irritation, vasodilation, hypersecretion, and nasal turbinate enlargement. The irritation occurs due to allergens, viruses, bacteria, pollutants, or other triggers that activate the immune system, causing the release of inflammatory mediators like histamine, leukotrienes, and cytokines. Vasodilatation increases blood flow, causing edema or fluid accumulation in the nasal tissues. Hypersecretion of mucus by goblet cells and submucosal glands attempts to flush out irritants but also adds to the obstruction of the nasal passages. Nasal turbinates can become swollen due to chronic inflammation or hormonal changes and reduce the nasal space [5]. The trigeminal nerve modulates sensory perception by transmitting airflow signals and localized pressure to the brain. However, psychological factors, environmental conditions, and medications could influence the perception of nasal congestion [1].

Various treatments are available over the counter (OTC) or by prescription to alleviate the nasal congestion associated with AR [6,7]. Phenylephrine is a sympathomimetic drug that acts on alpha/adrenergic receptors in the nasal blood vessels, causing vasoconstriction and reducing nasal congestion. Phenylephrine hydrochloride 10 mg (PE HCl 10 mg) is an oral decongestant marketed for OTC use in the United States for temporary relief from nasal congestion due to the common cold, AR, or other upper respiratory tract allergies and for quick relief from sinus congestion and pressure. However, some systemic reviews and meta-analyses have questioned its efficacy and safety compared to placebo [8]. Many studies that supported the original 1976 Food and Drug Administration (FDA) labeling for nonprescription use of phenylephrine did not find that PE HCl 10 mg is effective in the treatment of nasal congestion [8]. This systematic review aims to update the information on the efficacy of oral phenylephrine versus placebo on adults treating nasal congestion over the years. Considering this research, the most important in recent years due to the previous information gathered by the FDA, this research will try to demonstrate that a placebo is equal to or superior to taking phenylephrine.

## Review

Methods

The present study employed the Preferred Reporting Items for Systematic Review and Meta-Analysis (PRISMA) 2020 guidelines to conduct a comprehensive systematic review [9].

Searching Methods

Inclusion and exclusion criteria were used to select only high-quality studies for analysis. A rigorous exclusion criterion was applied to ensure the quality and relevance of the studies included in the analysis. Studies that did not relate to the use of oral phenylephrine for nasal congestion reported on animal models or did not contain original data. In addition, studies that were not available in full text or could not be obtained through interlibrary loans were excluded. We searched PubMed (Table 1), Cochrane (Table 2), and Scopus (Table 3) using Mesh and free-text terms related to our research question on 09/18/2023.

**Table 1:** Pubmed/Medline Search Specific search string used to identify relevant articles on Pubmed/Medline database. Search and extraction date 09/18/2023.

SEARCH	RESULTS
((phenylephrine[MeSH Terms]) OR (phenylephrine [Title/Abstract])) AND ((nasal decongestants[MeSH Terms]) OR (Nasal decongestants [Title/Abstract]))	149

**Table 2:** Cochrane Library Search Specific search string used to identify relevant articles on Cochrane Library. Search and extraction date 09/18/2023.

SEARCH	RESULTS
#1 MeSH descriptor: [Nasal Decongestants] explode all trees	242
#2 MeSH descriptor: [Phenylephrine] explode all trees	943
#3 Nasal decongestants	362
#4 Phenylephrine	2475
#5 (#1 OR #3) AND (#2 OR #4)	39

**Table 3:** Scopus Search Specific search string used to identify relevant articles on Scopus. Search and extraction date 09/18/2023.

SEARCH	RESULTS
Nasal Decongestant AND Phenylephrine	644

Types of Study

For our research, “The use and efficacy of Oral Phenylephrine versus placebo on adults treating nasal congestion over the years,” we conducted a systematic review of relevant studies published from 1998 to 2023, available in English. We meticulously screened and analyzed semi-randomized controlled trials (RCT), semi-randomized. This systematic review included studies that met the following inclusion criteria: RCT and case-control studies reporting the use and efficacy of oral phenylephrine versus placebo on adults treating nasal congestion. We excluded case reports, case series, meta-analyses, systematic reviews, dissertations, book chapters, protocol articles, reviews, news articles, conference abstracts, letters to the editor, editorials, and comment publications. Furthermore, we excluded studies that did not clearly describe their operationalization, duplicates, and those for which we could not obtain the necessary data or receive a response from the original author via email.

Types of Participants

This study has set specific participant selection criteria, including both genders. The focus will be on adult use of oral phenylephrine as a treatment for nasal congestion. Including only articles that report the use of oral phenylephrine and placebo for treating nasal congestion from inflammatory etiology. Excludes studies involving pediatric populations (under 18 years of age). The study aims to include a variety of participants to gain a better understanding of the intervention.

Types of Intervention

To be eligible for inclusion in this study, the selected research must evaluate the effect of oral phenylephrine. The interventions may include oral supplements or any other consumption way. The control group can receive no intervention, standard care, or alternative intervention. Excludes studies that do not involve the administration of oral phenylephrine in any of the subgroups or groups.

Outcomes

The outcomes to be measured include studies that report relevant outcomes changes explicitly in nasal congestion symptoms and Exclude studies that do not report relevant outcomes related to nasal congestion relief. The primary goal of this systematic review was to determine whether there is sufficient evidence that nonprescription oral phenylephrine is efficacious in relieving nasal congestion as measured by nasal airway resistance (NAR). Secondary objectives included examining the dose-response effects of oral phenylephrine, a review of efficacy as measured by patient-reported symptoms, and safety as measured by changes in heart rate and blood pressure [10].

Data extraction

Selection of Studies

Following an initial screening based on the title and abstract, two reviewers (JRFV and MMO) independently selected trials for inclusion in this review using predetermined inclusion and exclusion criteria. This search used Rayyan (Qatar Computing Research Institute, Qatar) to extract relevant data, and duplicates were filtered. Keywords were employed to highlight inclusion and exclusion criteria-related words on Rayyan [9].

Any disagreements about including studies were resolved through consensus and consultation with a third review author (ECM). Subsequently, a full-text analysis was conducted, with two reviewers (JRFV and SSK) independently selecting trials for inclusion in this review using predetermined inclusion and exclusion criteria. Disagreements about including studies were resolved through consensus and consultation with a third review author (ECM).

Data evaluation

Assessment of Risk of Bias in Included Studies

We evaluated the data using the criteria outlined in the Cochrane Handbook. To assess the quality of studies in the systematic review, we applied the Cochrane Risk of Bias (RoB) 2.0 tool [11], which examines potential bias in domains including selection, performance, detection, reporting, attrition, and other forms of bias for RCTs. Two independent reviewers evaluated the RoB in each study, considering the specific criteria and guidelines provided by the respective tools. Any discrepancies were resolved through discussion or consulting with a third, blinded reviewer as needed. The methodological components of the trials were assessed as having a low, high, or unclear risk of bias under the Cochrane Handbook for Systematic Reviews of Interventions [12]. Details of any down- or up-grading of the quality of evidence will be presented in the summary of findings table, providing transparency and explanations for the assessment of bias in each included study.

Results

**Figure 1:**
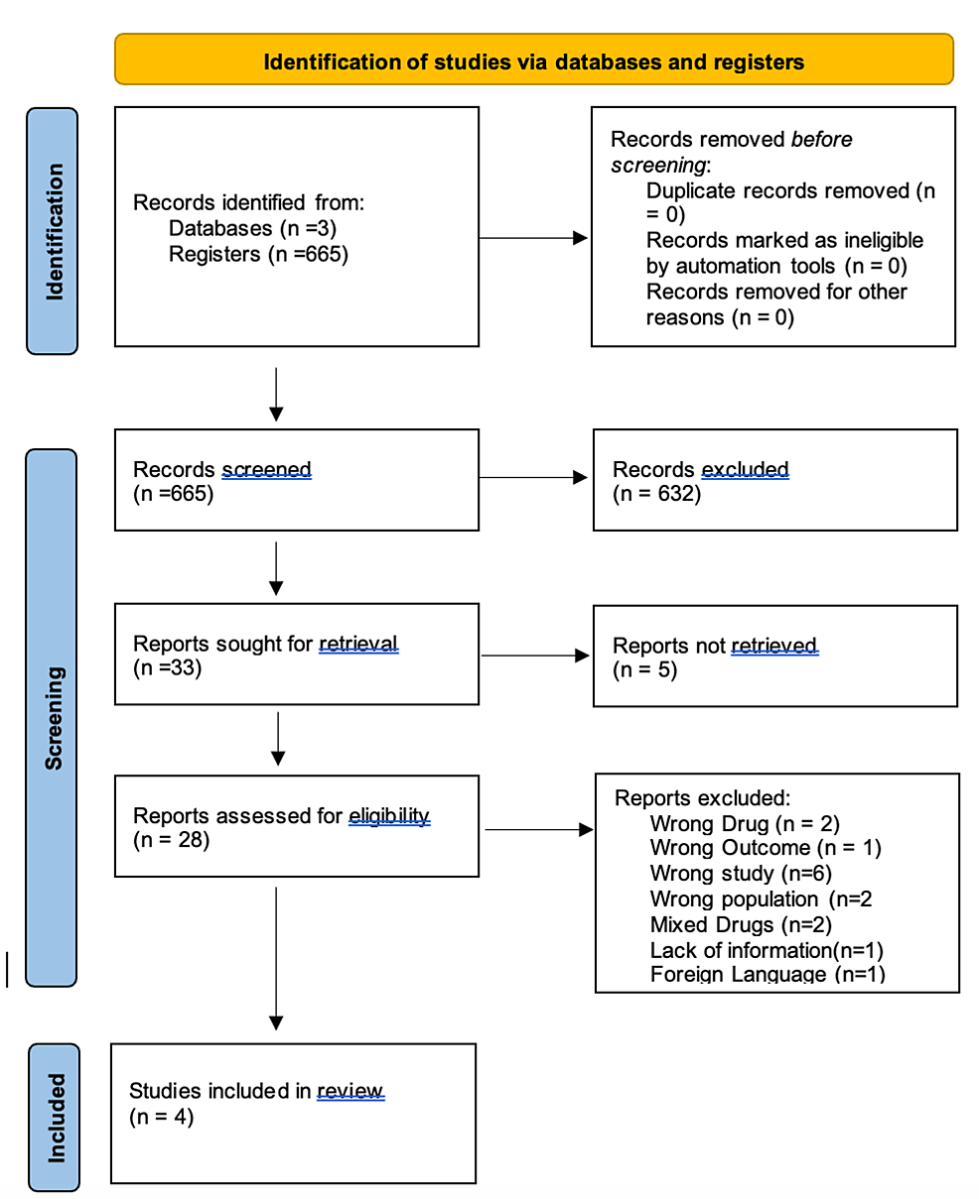
Study Flow Diagram Flow diagram (based on Preferred Reporting Items for Systematic Review and Meta-Analysis (PRISMA) statement) [8]. From the three different databases previously mentioned, we identified a total of 665 articles that were screened. Out of the 665 articles screened, 33 articles were sought for retrieval, and only 28 studies were assessed for eligibility. We concluded the assessment with only four articles included in this systematic review.

**Figure 2:**
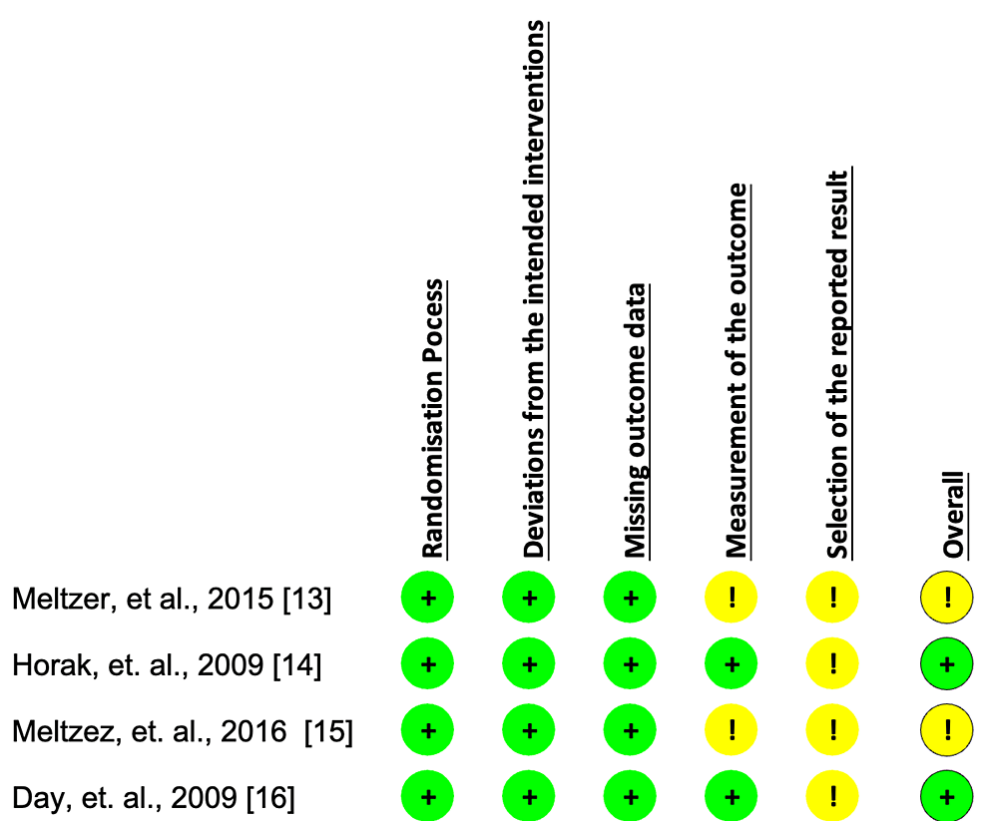
Risk of Bias Assessment This assessment was performed using the Risk of Bias 2.0 Tool By Cochrane for Randomized Control trials [11]. Risk of bias in each article. The first and third articles had an overall concern for bias, and the second and fourth articles had a low risk of bias. Meltzer et al., 2015 [13], Horak et al., 2009 [14], Meltzez et al., 2016 [15], Day et al., 2009 [16]

Study identification and selection across the database allowed us to narrow the pool of possible articles down to 665 articles. After a thorough examination, 0 duplicate articles were eliminated. Thirty-three publications were selected for further review after an initial screening of titles and abstracts, followed by the retrieval of complete texts. After determining the eligibility and quality of the full-text papers that had been shortlisted, four articles were selected for the review process. Figure 1 of the PRISMA flow chart depicts the studies' selection procedure [8], whereas Figure 2 shows the assessment of articles using the relevant Cochrane quality appraisal tools for eligibility [10].

The studies varied in design, including clinical, multicenter randomized, single-center, single-dose double-blind.

The quality of the studies was generally high according to the RoB 2.0 tool [11], with all studies being published in peer-reviewed journals from 1998 to 2023. However, four studies were evaluated with a total population of 1532 (Table 4). All studies had an oral phenylephrine intake of 10mg to 40 mg. All of the studies included in this research showed that phenylephrine was not significantly different from placebo. The main aim of all of the studies was to evaluate and draw any difference between using phenylephrine or placebo in relieving nasal congestion. Measuring mean nasal congestion in all of the studies that stated that phenylephrine was not significantly better than placebo, two out of the four studies included one or more drugs additional to phenylephrine. The addition of the drug did not jeopardize the placebo. The results narrow down or indicate that nasal congestion is a complex condition with various underlying pathophysiological mechanisms.

**Table 4:** General Outcomes of Included Studies PE: phenylephrine; PHE-MR: phenylephrine hydrochloride modified-release; PE HCl: phenylephrine hydrochloride; HR: heart rate; SBP: systolic blood pressure

Author, year	Study Design	Population Characteristics	Sample Size	Intervention	Result	Comments
Meltzer et al., 2015 [13]	Multicenter, randomized, phase 2, parallel, 5-arm, open-label, placebo-controlled dose-ranging trial of treatment for seven days	Healthy participants over the age of 18 years with documented or patient-reported history of SAR due to spring pollen within the last four years AND symptoms over at least the last two spring allergy seasons	539	Patients randomized into 5 groups- PE HCl 10 mg intermediate release tablets at fixed doses of 10, 20, 30, 40 mg and placebo	Phenylephrine was not significantly different from placebo in any of the active treatment groups for the primary endpoint, mean change from baseline in reflective nasal congestion scores. All secondary endpoints were not statistically significant, except for some on day 6 favoring PE, namely mean change in evening reflective nasal congestion scores (P .0188), the response rate (P .031) and mean change in daily reflective nasal congestion scores (P .0201), all for the 20 mg PE HCl group compared with placebo.	Phenylephrine was not more effective than placebo at doses of 10 to 40 mg every 4 hours. Doses of up to 30 mg PE HCl are well tolerated. Dose-related increases in SBP and dose-related decreases in HR were noted on day 1 of treatment, which were resolved by day 8.
Horak et al., 2009 [14]	Single-center, randomized, placebo-controlled, 3-way crossover study	Grass-sensitive patients	39	Patients were dosed with immediate-release formulations of phenylephrine, 12 mg, pseudoephedrine, 60 mg, as a control, or placebo.	Phenylephrine was not significantly different from placebo in the primary endpoint, mean change in nasal congestion score at more than 6 hours (P .56), whereas pseudoephedrine was significantly more effective than both placebo (P .01) and phenylephrine (P .01).	During a 6-hour observation period, a single dose of pseudoephedrine but not phenylephrine resulted in significant improvement in measures of nasal congestion. Neither phenylephrine nor pseudoephedrine had an effect on nonnasal symptoms.
Meltzez et al., 2016 [15]	Multicenter, randomized, double-blinded, placebo-controlled, 2-arm, parallel-group phase 3 trial.	Eighteen years and above with documented or self-reported history of AR caused by fall pollen within the past four years or symptoms thereof for at least the two previous fall allergy seasons or a documented skin prick test reaction to fall pollen allergens or intradermal test reaction within the past four years.	575	PEH-MR or placebo every 12 hours for seven days	No significant beneficial difference was detected between PEH-MR and placebo for the primary endpoint (PEH-MR, mean 0.394, SD 0.4880; placebo, mean 0.412, SD 0.5383; P ¼ .2655). Likewise, no significant differences were observed for most secondary endpoints or quality of life. Overall, 89 of 575 patients (15.5%), equally distributed between the PEH-MR and placebo groups, experienced at least one treatment-emergency adverse event.	No specific effort was made to exclude patients with upper respiratory tract infections (URIs) in this study.
Day et al., 2009 [16]	Phase 3, single-dose, double-blind, double-dummy, randomized, placebo-controlled, 3-arm, parallel-group, single-center study	Patient with at least minimum symptoms during ragweed pollen exposure	379	Single dose of Loratadine-Montelukast (10mg/10mg), placebo and phenylephrine(10mg)	During the first 6 hours after treatment (primary endpoint), loratadine-montelukast treatment resulted in greater improvement in the mean nasal congestion score vs placebo (P .007) and phenylephrine (P .001). Loratadine-montelukast was more effective than placebo (P .02) and phenylephrine (P .002) in relieving total symptoms, nasal symptoms, and nonnasal symptoms and in improving peak nasal inspiratory flow. There were no statistically significant differences between phenylephrine and placebo for any measures. Fewer patients in the loratadine-montelukast group (3.9%) reported adverse events than in the phenylephrine (7.9%) and placebo (7.1%) groups; most adverse events were mild or moderate.	There was no statistically significant difference between phenylephrine and placebo.

Discussion

Nasal congestion is an often-encountered symptom in clinical practice that can be quite bothersome to the inflicted individual [16]. Out of the multiple therapeutic approaches to this clinical conundrum, our study aimed to assess the efficacy and safety found over the years by using oral phenylephrine versus the administration of a placebo and consequently evaluating the degree of nasal congestion relief obtained in the affected individuals. Our results suggest that phenylephrine is not significantly different from placebo, for the primary objective focused on the reduction of NAR in four out of the five studies included in this systematic review [13,15,16]. In one out of the four included studies, there is some concern about bias [15]. While the remaining studies have a low risk of bias [14,16]. This leads us to believe that the conclusions that we may draw from this analysis are reliable. No discernible differences were noted between male and female participants subjected to the treatment methods in the above studies. Measuring the decreased levels of NAR over 120 min by using oral phenylephrine 10, 15, and 25 mg did not result in solid evidence that can support the efficacy of oral phenylephrine in treating NAR. Safety was assessed by measuring vitals which included blood pressure and heart rate changes after phenylephrine dosing in four out of five studies. An electrocardiogram (ECG) was used as an additional indicator for safety in one of the studies, which did not show any clinically significant differences in any of the treatment groups [6]. With the subsequent extraction of the data obtained for the consideration of the symptoms of congestion relief and the symptoms developed after the administration of each phenylephrine dose, adverse events were also noted in all the studies as a measure of safety. The most common adverse event encountered was headache, seen in two of the four studies [15,16]. No life-threatening adverse events were seen in any of the studies included in this systematic review. However, in the first study, one participant from the 40 mg phenylephrine dose treatment group experienced chest and lower jaw pain, which promptly resolved on withdrawal of phenylephrine [13]. The frequency of side effects was noted to increase with increasing doses of phenylephrine, with the highest dose in the study being 25 mg, and as much as 81.3% of the treatment group experiencing at least one side effect. In the four studies, it was revealed that in the phenylephrine treatment group, three participants developed complaints of epistaxis, and two participants experienced severe AEs in the posttreatment observation group, which included severe headache and severe sinus headache for two days with nausea and vomiting for one day, respectively [16]. For the most part, no treatment differences were observed in the vital signs measured except in one study, which demonstrated dose-related increases in systolic blood pressure and dose-related decreases in heart rate on day 1, all of which resolved by day 8 of the study [13].​​

In the first study [13], while there was no statistically significant difference between phenylephrine and placebo for the primary objective, a few of the secondary endpoints, namely change from baseline for the evening reflective nasal congestion scores, the response rate and the change from baseline for daily reflective nasal congestion scores supported phenylephrine over placebo, all at the 20 mg dose of phenylephrine. In the fourth study, conducted to determine the efficacy of phenylephrine hydrochloride modified-release (PEH-MR) tablets over that of placebo for nasal congestion, PEH-MR was found to be not significantly better than placebo for the primary endpoint, as well as most of the secondary endpoints and overall quality of life [15]. It is hypothesized that the decrease in efficacy of phenylephrine can be attributed to the low level of plasma concentration achieved by oral formulations of phenylephrine [13], but we see here even with the modified-release tablets, which are tailored to maintain drug plasma concentrations at optimal levels, phenylephrine still shows a lack of efficacy when compared with placebo.

The results build on the existing theory that phenylephrine is not consistent in bringing about relief from nasal congestion when administered orally, at the dosages usually found in OTC products. The apparent lack of efficacy of phenylephrine could be attributed to its low bioavailability (38%) [13] when given as oral preparations, due to which the drug is not present at optimal concentrations in the plasma to have a significant effect. The highest dose noted in this study at which the efficacy of phenylephrine was tested was 40 mg, with which patients experienced multiple AEs, the most common of which were gastrointestinal side effects. However, doses of up to 30 mg were well tolerated.

In our systematic review of oral phenylephrine's efficacy in treating nasal congestion, we encountered contrasting findings compared to two prior systematic reviews and meta-analyses conducted in 2007 [17,18]. Those earlier studies suggested that oral phenylephrine was an effective treatment for nasal congestion associated with the common cold. However, we identified significant limitations in the earlier research that prompted our own investigation. A primary concern with the previous reviews was that they predominantly included articles published before 1998. This raised doubts about the applicability of their conclusions in the context of contemporary medical practices, which have evolved considerably. Furthermore, the earlier reviews did not assess the risk of bias in the included articles, a critical step in evaluating the quality of evidence. This omission could have introduced bias into their conclusions, necessitating a fresh review with more recent research.

To address these concerns and provide up-to-date evidence with a rigorous methodology, we limited our systematic review to more recent articles. This approach aimed to ensure that our findings accurately reflected the current state of knowledge regarding oral phenylephrine's efficacy in treating nasal congestion. Another relevant aspect we considered was the regulatory perspective on medications. The FDA has raised concerns about the effectiveness of specific drugs, including oral phenylephrine as a decongestant and guaifenesin as an expectorant [19]. Given these regulatory doubts, it is crucial to critically assess available evidence and reconsider the recommendations for these medications in specific medical contexts.

Additionally, while our systematic review focused on the adult population, there is a need for a dedicated systematic review for the pediatric population. This is because medication responses can vary between adults and children. However, it is essential to note that the pharmacokinetics and metabolism of oral phenylephrine are similar in both populations [20]. Consequently, our findings are likely applicable to both adults and children.

Limitations

Although we have strived to provide a reliable and thorough analysis of our study objectives, our review does have its constraints. Our review was focused on articles published exclusively in the English language, and only those that included clinical trials, excluding a vast array of other publication types. Though it was not our intent, through such exclusion, a substantial amount of data that may have otherwise contributed to our interpretations may have been neglected. Our study also was not subjected to distinct criteria regarding the drug dosage, or the requirement of only a single dose of phenylephrine. Studies that included a comparison between phenylephrine and other drugs were not excluded either. This may have influenced our interpretation of the selected articles.

The above results show insufficient evidence to deem oral phenylephrine efficacious for nasal congestion, which should be considered when prescribing decongestants for nasal obstruction in clinical practice.

## Conclusions

In conclusion, the systematic review aimed to assess the efficacy and safety of oral phenylephrine versus placebo in treating nasal congestion. We can conclude that oral phenylephrine is not different from placebo in relieving nasal congestion, as measured by NAR. This implies that the OTC use of phenylephrine for nasal congestion may not be supported by strong evidence. This review also highlighted some safety concerns, with adverse events such as headache, dry mouth, and other mild to moderate side effects being reported. However, there were no life-threatening adverse events, and the safety profiles were generally similar between phenylephrine and placebo.

It's essential to consider these findings when recommending or using oral phenylephrine for nasal congestion, and clinicians may want to explore alternative treatment options. The limitations of this review, such as the exclusion of non-English articles and varying dosages, should also be kept in mind when interpreting these results. Overall, further research may be needed to clarify the role of oral phenylephrine in managing nasal congestion.
